# Prevalent coinfection and associated factors for Hepatitis B, Hepatitis C, and Human Immunodeficiency Virus in patients submitted to renal replacement therapy: A cross-sectional study of 21 dialysis units in the State of Mexico

**DOI:** 10.1371/journal.pone.0275238

**Published:** 2022-12-01

**Authors:** Silvia Palomo-Piñón, Neftali Eduardo Antonio-Villa, Luis Rey García-Cortés, David Rojano-Mejía, Paula González-Palomo, Marilin Victoria Martínez-Olivares, Leopoldo Santillán-Arreygué, Olga Margarita Bertadillo-Mendoza, Oliva Mejia-Rodriguez, Abraham Santos Ontiveros, Maria de los Angeles Dichi-Romero, Blanca Estela Herrera-Morales, Berenice Serafín-Méndez, Flor Araceli Nava-Ayala, Delfino Torres-Valle, Francisco Medrano-Lopez, Tabata Gabriela Aguinao-Velazquez, Antonio Aguilar de los Santos, Alfonso Hernandez Cruz, Maria Adriana Cruz-Arce, Marcos Sebastian Pineda Espinosa, Laura Mejia-Dominguez

**Affiliations:** 1 Coordinación de Investigación en Salud, Instituto Mexicano del Seguro Social, Ciudad de México, México; 2 Programa de Posgrado en Ciencias Médicas, Odontológicas y de la Salud, Universidad Nacional Autónoma de México, Ciudad de México, México; 3 MD/PhD PECEM (Program), Facultad de Medicina, Universidad Nacional Autónoma de México, Ciudad de México, México; 4 Coordinación Auxiliar Médica de Investigación en Salud, Instituto Mexicano del Seguro Social, Estado de México Oriente, México; 5 Coordinación de Investigación en Salud, Centro Médico Nacional Siglo XXI, Instituto Mexicano del Seguro Social, Ciudad de México, México; 6 Instituto de Oftalmología FAP Conde de Valenciana, Ciudad de México, México; 7 Subjefatura de Enfermeria, UMAE Hospital De Especialidades Dr. Antonio Fraga Mouret, Centro Medico Nacional La Raza, Ciudad de México, México; 8 Órgano de Operación Administrativa Desconcentrada de Coahuila, Instituto Mexicano del Seguro Social, Ciudad de México, México; 9 Planeación y Enlace Institucional, Instituto Mexicano del Seguro Social, Estado de México Oriente, México; 10 División de Investigación Clínica, Centro de Investigación Biomédica de Michoacán, Instituto Mexicano del Seguro Social, Morelia, Michoacán, México; 11 Servicios Médicos y de Equipamiento SA de CV, Ciudad de México, México; 12 Jefatura de Servicios de Prestaciones Médicas, OOAD Regional Estado de Mexico Oriente, Instituto Mexicano del Seguro Social, Estado de México, México; 13 Hospital General Regional 196, Instituto Mexicano del Seguro Social, Estado de México, México; 14 Hospital General de Zona 57, Instituto Mexicano del Seguro Social, Estado de México, México; 15 Hospital General de Zona 68, Instituto Mexicano del Seguro Social, Estado de México, México; 16 Hospital General Regional 71, Instituto Mexicano del Seguro Social, Estado de México, México; 17 Hospital General Regional 72, Instituto Mexicano del Seguro Social, Estado de México, México; 18 Hospital General Regional 76, Instituto Mexicano del Seguro Social, Estado de México, México; 19 Hospital General de Zona 98, Instituto Mexicano del Seguro Social, Estado de México, México; 20 Hospital General de Zona 197, Instituto Mexicano del Seguro Social, Estado de México, México; 21 UMF/UMMA 198, Instituto Mexicano del Seguro Social, Estado de México, México; 22 Hospital General Regional 200, Instituto Mexicano del Seguro Social, Estado de México, México; 23 Hospital General Zona 53, Instituto Mexicano del Seguro Social, Estado de México, México; Instituto Nacional de Geriatria, MEXICO

## Abstract

**Background:**

Chronic kidney disease (CKD) predispose to viral coinfections in patients submitted to renal replacement therapy (RRT); nevertheless, few reports have been performed to elucidate the current epidemiology within this population in Mexico.

**Aim:**

To estimate the prevalence of HBV, HCV, and HIV coinfection and to explore factors associated with prevalent coinfection in patients living with renal failure undergoing to RRT.

**Methods:**

A multicenter cross-sectional recruitment across 21 units at the Mexican Institute of Social Security (IMSS) at the State of Mexico was performed during 2019. A standardized clinical questionnaire was performed to elucidate individual and relatives-related conditions. A treatment facility questionnaire was applied to the chief responsible of each unit to explore treatment facility variables. Serological testing, clinical, biochemical, and anthropometrical parameters were extracted from clinical records.

**Result:**

In 1,304 patients (57.5% male, mean age 45.5 (SD: 15.6) years, and 95.8% in hemodialysis), the prevalence of any viral coinfection was 3.14% (95% CI: 2.32%-4.23%). The highest viral coinfection prevalence were for HCV, HBV, and HIV, in which men and subjects diagnosed after 2010’s had the highest rates. We identify that being submitted to peritoneal dialysis, being treated in a surrogated dialysis center and living with a close relative with prior hepatitis coinfection were associated factors for any viral coinfection.

**Conclusion:**

In patients submitted to RRT, the prevalence of viral coinfection remains high compared with general population. Screening strategies, medical awareness and targeted public healthcare policies should prioritize better care practices within patients submitted to RRT in Mexico.

## Introduction

Chronic kidney disease (CKD) is a chronic health condition with a high morbidity and mortality worldwide [1]. Overall, people living with CKD in low-and-middle income countries (LMIC) experience a high burden of CKD due to related complications that includes coinfections of viral diseases [2]. Previous studies have demonstrated that undergoing to renal replacement therapy (RRT) increases the odds to acquire Hepatitis B (HBV), Hepatitis C virus (HCV) and Human Immunodeficiency Virus (HIV) in patients living with CKD. This association has been documented since 1990’s, as patients submitted to RRT had increased exposure to blood transfusions and hemodialysis interventions [3–5]. Since then, diverse global efforts have been performed to create public-health strategies to mitigate the rate of HBV, HCV and HIV coinfection [6].

Mexico represents a challenging scenario, since a high proportion of patients living with CKD and undergoing to RRT are treated in specialized care-centers located within the centralized urban region of our country [7]. Although HBV, HCV, and HIV are considered infectious diseases of mandatory notification in Mexico, there are few epidemiological reports that have sought to evaluate the current status within patients undergoing RRT [8, 9]. Furthermore, the previous evidence in our country is restricted to monocentric studies or based their estimations on clinical self-report instead of serological testing. Hence, there is a need to better comprehend the current state of HBV, HCV, and HIV coinfection within this high-risk group to strength screening strategies and promote better care practices within the population living with CKD in Mexico.

Therefore, the objectives of this study were 1) to estimate the prevalence of HBV, HCV and HIV coinfection and 2) to explore factors associated with prevalent coinfection in patients living with renal failure undergoing to RRT in 21 dialysis units in the State of Mexico. We hypothesize that HBV, HCV, and HIV coinfection have decreased compared to previous reports among patients submitted to RRT due to mandatory and systematic screening that every patient needs before being submitted to any dialysis units in Mexico.

## Material and methods

### Study design and settings

We performed a multicenter cross-sectional study design in 21 dialysis units of the Mexican Institute of Social Security (IMSS) at the State of Mexico. We apply a clinical questionnaire in adult patients living with renal failure submitted to RRT and a second questionnaire of treatment facility conditions to the chief-staff of each dialysis unit during the period of January to December 2019. From our sample of adult patients, we search in their medical records for previously reported serological testing for HBV, HCV, and HIV. We excluded patients without complete clinical records files or subjects without IMSS affiliation number. All the information of the participants was anonymized, and a consecutive identification number was given to each participant. All patients signed a consent form before the clinical questionnaire was applied. The Investigation and Ethics Review Board of the IMSS approved this study under the protocol number R-2017-785-081.

### Outcome and exposure variables definitions

Our main analyses focused on the assessment of prevalent HBV, HCV and HIV coinfection defined as a case with positive antigen surface laboratory testing, being treated with any antiviral regime against HBV, HCV and HIV or had a history of self-reported viral coinfection. Diagnostic of each viral coinfection was homogenized for the 21 dialysis clinics according to the IMSS clinical guidelines [10–12]. Then, to evaluate the factors associated with prevalent coinfection we explore two main groups of exposure variables according to two aggregation levels:

- Individual level factors–the sociodemographic, clinical conditions, personal habits, and familiar-related conditions were explored as the individual probability of having prevalent coinfection.
- Treatment facility factors–we aggregate the number of coinfection cases by the 21 clinical facilities. Then, we explore factors using the prevalence rates standardized by the number of total of patients submitted to RRT within each clinical facility.

### Clinical questionnaire assessment

Our clinical questionnaire evaluated sociodemographic, clinical variables, personal habits, and familiar-related conditions. The anthropometrical and biochemical information was extracted from medical records. The questionnaire was applied by previously trained nurse personnel that applied individually to each patient our designed questionnaire. Further details of the training methods and complete questionnaire are presented in S1 File. The following variables were assessed in our questionnaire:

- Sociodemographic variables: Included age, sex, address of actual residence, registered dialysis unit, and social security number affiliation. Only age and sex are displayed in our results.
- Clinical conditions: This section included self-reported comorbidities, time since RRT initiation, current medication use, and renal failure etiology. The time of since RRT was defined using the date of the first dialysis in our medical units up to the date of application of our questionnaire.
- Personal habits: Evaluated using history of blood transfusion before 1995, history of surgical interventions, self-administration of intravenous illegal drugs, high-risk sexual activity (defined sexual intercourse with five or more different partners in one year [13]), use of acupuncture, self-reported tattoos, piercing and other perforations, as well as self-reported history of vaccination against HBV.
- Familiar related conditions: We asked whether the patient was living with a coinfected relative, the time since the relative coinfection diagnosis and its reported treatment, and whether any relative have previous vaccination against HBV.
- Anthropometric assessment: Weight and height were obtained from the clinical record of each patient. Body mass index (BMI) was calculated as weight in kg divided by the squared product of height in meters.
- Biochemical parameters: Included serum hemoglobin, albumin, creatinine, blood urea, electrolytes, and residual diuresis collected under 24 hours. The KT/V ratio was also included. These parameters were obtained from the clinical records of the last three months prior to our questionnaire assessment. Liver function enzymes were obtained within a period of three month after the coinfection diagnosis from clinical records.

### Questionnaire of clinical facility

A questionnaire of clinical facility conditions was applied to the chief responsible of each clinical facility included in our study. The questionnaire included whether each clinic was considered as an outside management clinic (surrogated dialysis unit), had mandatory serological testing policy prior to RRT, reuses dialysis filters for hemodialysis, performed monthly fumigation, cleaning, and disinfection of clinical facilities, and whether the involved healthcare workers had a history of HBV coinfection and had mandatory policy against HBV vaccination.

### Sample size estimation

We estimated a sample size according to the methods reported by Charan J et. al. [14]. We assess sample size estimations to observe seroprevalence for HCV and HBV based on the previously published works by Paniagua R et. al [9] and Méndez-Sánchez N [15]. We required to test a minimum of 562 patients to meet the requirements of the present study. Further details of this estimation are presented in S1 File.

### Statistical analysis

Continuous data are shown as means (standard deviation) or medians (interquartile range) according to their distribution evaluated through the Anderson-Darling normality test. Categorical variables are presented in absolute frequency and absolute percentage. All statistical analyses were performed using R Studio (Version 4.1.0). A value of p <0.05 was considered as our statistical significance threshold.

### Missing variables

We assessed and imputed missing biochemical values using multiple chained equations under the assumption that data was missed completely at random using the *mice* R Package (Version 3.14.0) [16]. We multiply 5 imputed datasets for a maximum of 5 iterations combined using Rubin’s rules. Detailed results of imputed variables are presented in S1 File.

### Prevalent coinfection estimation

We estimate the overall prevalence of HBV, HCV, and HIV coinfection along with its 95% confidence intervals (C.I.) using the “Wilson” method [17]. We further made stratifications by sex and decades of coinfection diagnosis (<2000’s, 2000 to 2010 and >2010’s). Confidence intervals for the prevalent coinfection rate were estimated using the *epiR* package (Version 2.0.3) [18].

### Factors associated with viral coinfection

Individual level factors associated with prevalent coinfection were explored using binomial logistic regression models. The independent variables were established to be sociodemographic, clinical conditions, personal habits, and familiar-related conditions. Treatment facility factors were explored using Poisson regression models. We considered the number coinfected cases as our dependent variable and the number of registered patients since the clinic’s opening as our model offset. Both odds ratio and risk ratio plots were created using the *jtools* package (Version 2.1.4) [19]. To obtain the model contribution for the percentage of variance explained by each model, we extract the McFadden R^2^. Models’ diagnostic was assessed using the Bayesian Information Criteria (BIC).

## Results

**Fig 1:**
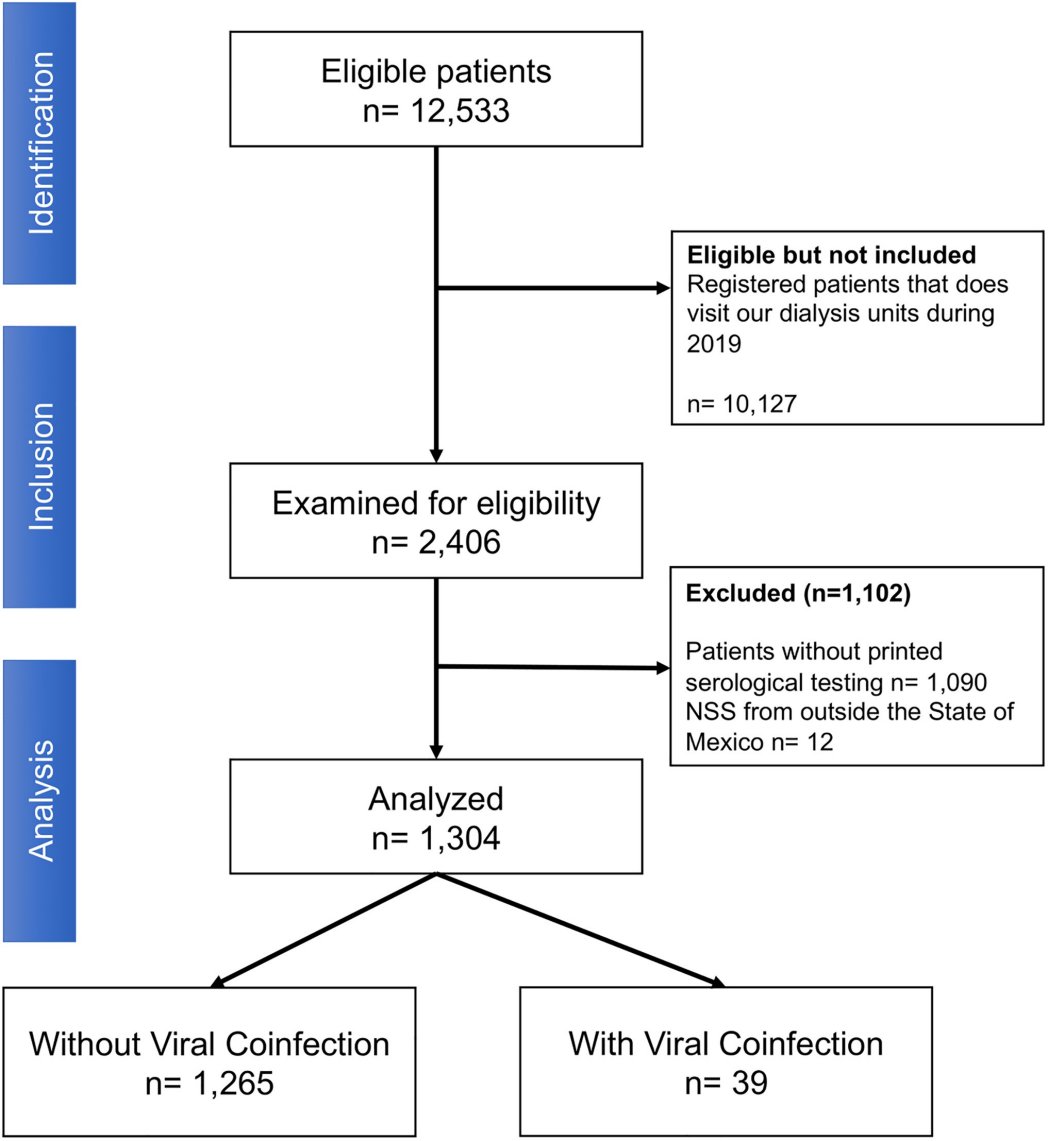
STROBE flow-chart of our studied population submitted to RRT in our 21 dialysis units in the State of Mexico.

We recorded 12,533 patients that underwent to RRT in all the 21 dialysis clinics since their opening. From this population, we apply our questionnaire in 2,406 during our study period, of which we excluded 1,102 due to incomplete clinical records or unavailable serological tests to explore coinfection of HBV, HCV, and HIV (Fig 1). Our final sample size was comprised of 1,304 patients. Complete clinical characteristics are presented in Table 1. Briefly, we had a male predominance (57.5%), with a mean age of 45.5 (SD: 15.6) years, in which hemodialysis (95.8%) was the main dialysis modality and only 4.2% were submitted to peritoneal dialysis. The median time of RRT was 3.1 (IQR: 1.3–5.7) years. We observed a high proportion of our population living with arterial hypertension (75.9%), anemia (37.8%), diabetes (33.8%), and overweight (28.3%). The leading etiology of renal failure was attributable to diabetes (25.3%). Although 77.7% remained as unclassified etiology.

**Table 1:** Sociodemographic and clinical characteristics of patients submitted to renal replacement therapy from the Metropolitan Area of Mexico.

Parameter	All-Population (n = 1,304)	Without Any-Coinfection (n = 1,265)	Any-Viral Coinfection (n = 39)	P value
Men (%)	750 (57.52)	724 (57.23)	26 (66.67)	0.235
Age (years)	45.4 (SD: 15.59)	44.5 (SD: 15.08)	44.13 (SD: 13.32)	0.864
Hemodialysis (%)	1249 (95.78)	1212 (95.81)	37 (94.87)	0.981
Time since RRT (Years)	3.05 (1.25–5.69)	3.08 (1.25–5.7)	4.34 (1.85–8.17)	0.038*
Residual Uresis (ml/day)	0 (0–200)	0 (0–200)	200 (0–700)	0.046*
KT/V	1.1 (1.0–1.69)	1.16 (1.0–1.7)	1.3 (1.0–1.65)	0.461
Body Mass Index (kg/m^2)	24.37 (21.75–27.32)	24.09 (21.45–27.12)	22.66 (20.11–26.62)	0.106
Underweight (%)	56 (4.29)	52 (48.77)	4 (48.77)	0.981
Normal weight (%)	638 (48.93)	617 (8.7)	21 (8.7)	0.875
Overweight (%)	369 (28.3)	359 (48.77)	10 (48.77)	0.591
Obesity (%)	130 (9.97)	127 (8.7)	3 (8.7)	0.622
Diabetes (%)	441 (33.82)	430 (33.99)	11 (28.21)	0.515
Arterial Hypertension (%)	990 (75.92)	959 (75.81)	31 (79.49)	0.864
Previous CVD (%)	131 (10.05)	128 (10.12)	3 (7.69)	0.774
Anemia (%)	493 (37.81)	472 (37.31)	21 (53.85)	0.048*
Retinopathy (%)	239 (18.33)	237 (18.74)	2 (5.13)	0.028*
Current Smoking (%)	31 (2.38)	31 (2.45)	0 (0)	0.615
Alcoholism (%)	19 (1.46)	19 (1.5)	0 (0)	0.656
Neurological Impairment (%)	34 (2.61)	237 (18.74)	2 (5.13)	0.626
Lupus (%)	22 (1.69)	189 (14.94)	3 (7.69)	0.981
Diabetic Nephropathy (%)	330 (25.31)	318 (25.14)	12 (30.77)	0.466
Unclassified Nephropathy (%)	974 (74.6)	947 (74.8)	27 (69.2)	0.123

*Abbreviations*: RRT = renal replacement therapy; CVD = cardiovascular disease.

SD = Standard deviation

### Characterization of coinfected patients

We observed that 41 (3.72%) patients were diagnosed with any viral coinfection during our study period. Seventeen patients were diagnosed with HBV, 22 with HCV, and 5 with HIV; 2 patients were coinfected with HBV and HCV and one with HCV and HIV, which were excluded from the prevalent coinfection analysis. We observed that patients with any viral coinfection trend to had higher time since RRT (median 4.34; IQR: 1.85–8.17 years), and a more increased residual diuresis (median 200, IQR: 0–700 ml/day) along with a higher proportion of anemia (54.8%) compared with patients without any-coinfection (Table 1). Regarding medication and biochemical characteristics, we observed a decreased proportion of iron supplementation (12.8%) and lower levels of direct bilirubin (median: 0.09, IQR: 0.08–0.1 mg/dl) compared to patients without prevalent coinfection (S1 File).

### Prevalent coinfection rate in our study population

**Fig 2:**
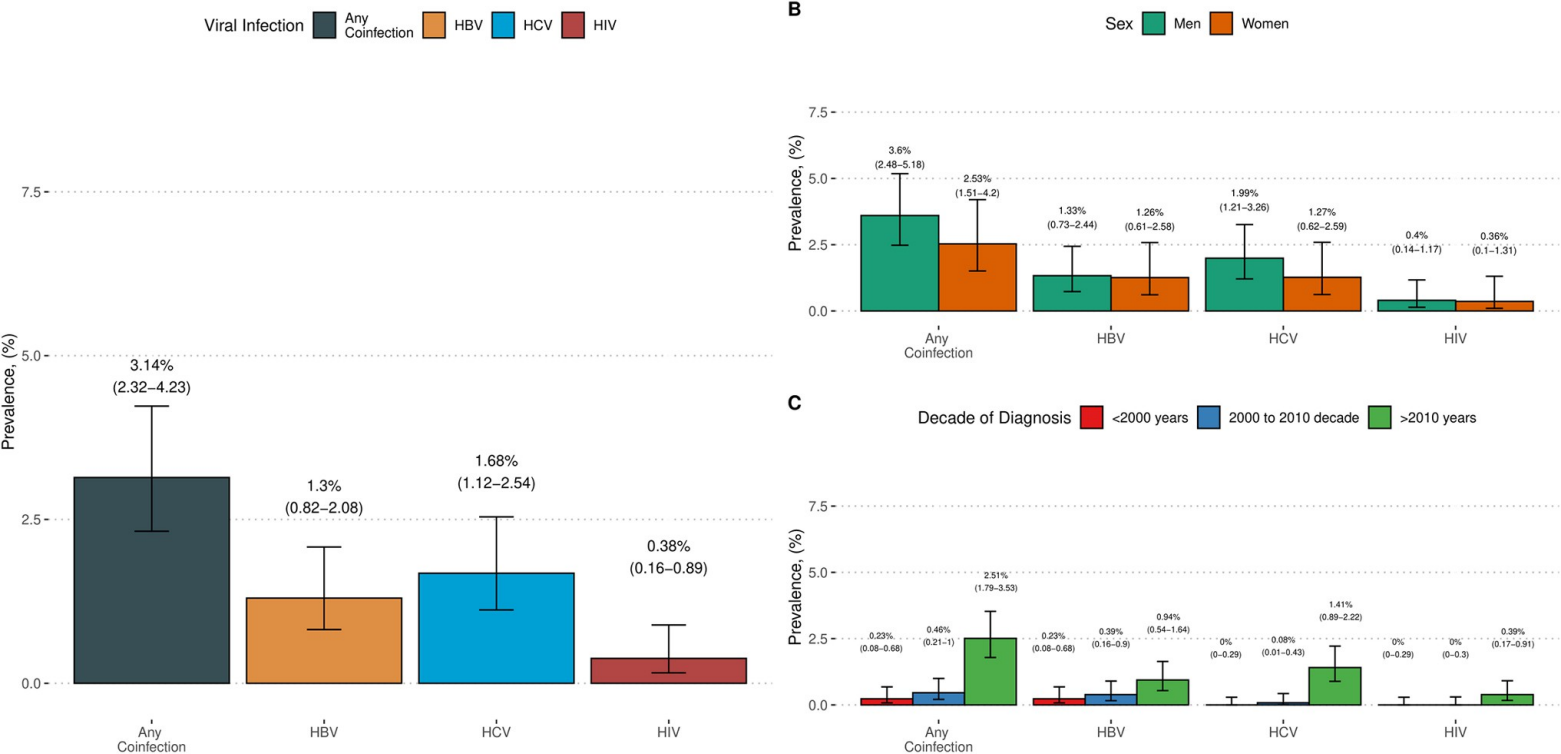
Estimated prevalence with 95% confidence intervals for any coinfection associated with HBV, HCV, and HIV. *Abbreviations*: HBV = Hepatitis B Virus, HCV = Hepatitis C Virus; HIV = Human Immunodeficiency Virus. Confidence intervals were calculated using the “Wilson” method.

We estimated a prevalence of any viral coinfection of 3.14% (95% CI: 2.32%-4.23%) in our study population. The highest viral coinfection was HCV, with an estimated prevalence of 1.68% (95% CI: 1.12–2.54%), followed by HBV with 1.3% (95% CI: 0.82%-2.08%) and HIV with 0.38% (95% CI: 0.16%-0.89%). After sex stratification, we observed that men trend to had increased prevalence of any type of coinfection (3.6%, 95% CI: 2.48%-5.18%), particularly driven by HCV (1.9%, 95% CI: 1.21%-3.26%). Then, we stratify for decades since viral diagnoses and observed an increasing trend of coinfection where we observed a 2.5% (95% CI: 1.79%-3.53%) prevalence in diagnoses years after 2010, followed by 0.46% (95% CI: 0.21%-1.0%) for the years of 2000–2010 and a 0.23% (95% CI: 0.08%-0.68%) for years prior to 2000 (Fig 2).

### Associated factors related to coinfection

**Fig 3:**
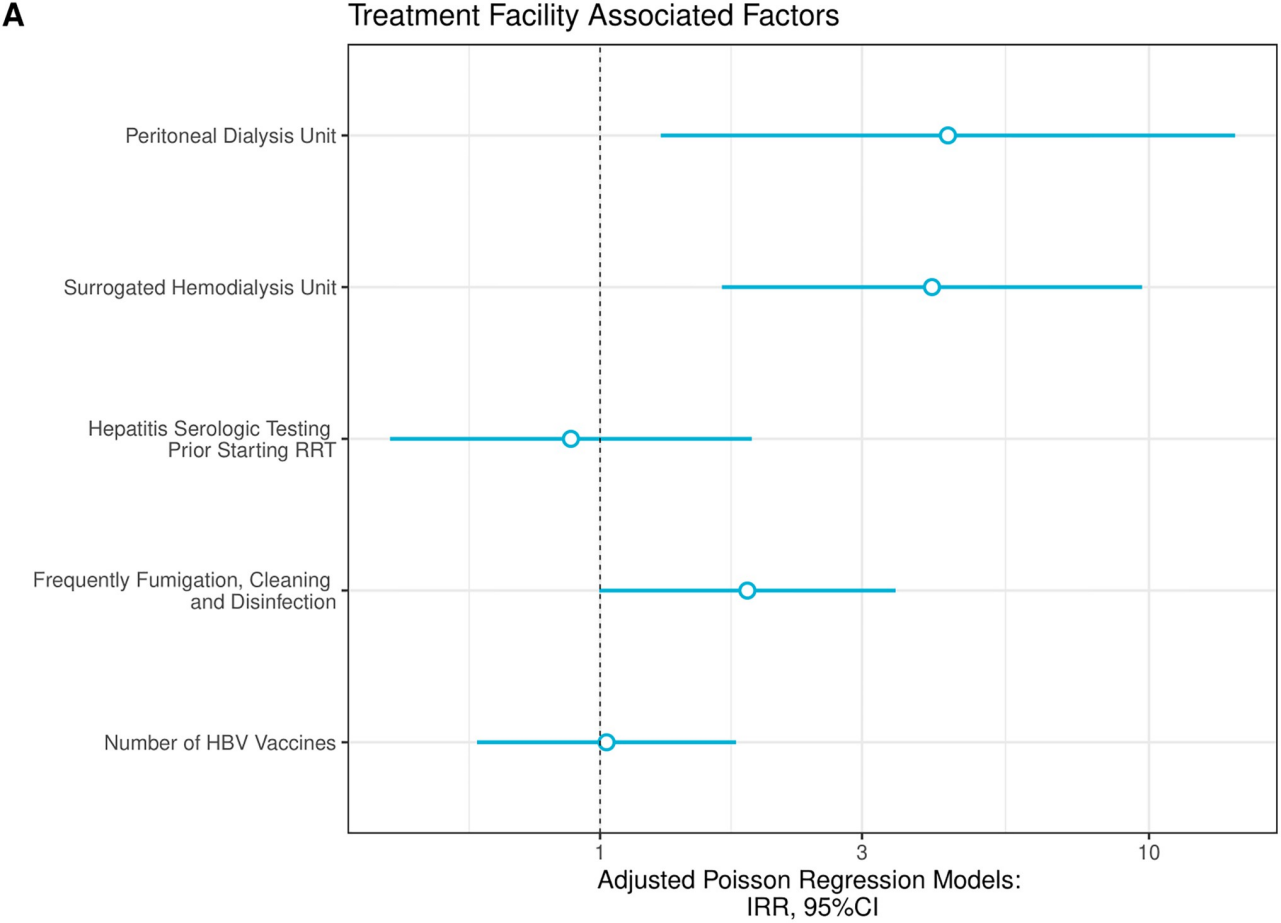
Poisson regression models to assess treatment facility factors related to any viral coinfection (A).

Regarding associated factors related to clinical facility, we observed that patients submitted peritoneal dialysis (RR: 4.30; 95% CI: 1.29–14.35) had increased risk-ratio to have any type of viral coinfection compared with subjects submitted to hemodialysis units. Furthermore, patients that were treated within surrogated dialysis centers had 4.2-fold-increased rate (95% CI: 1.67–9.71, p = 0.02) compared with those treated in affiliated dialysis centers (Fig 3). We observed in our logistic regression model that the only associated factors was living with a coinfected relative, which conferred a 66% (95%CI: 1.01–7.77, p = 0.07) increase in odds to have any type of viral coinfection compared with patients without this condition.

## Discussion

In this study, we performed a cross-sectional recruitment of 1,304 patients to assess the prevalence of HBV, HCV and HIV coinfection in patients living with renal failure submitted to 21 dialysis units from the Mexican Institute of Social Security in Mexico. We observed that approximately 3.14% (95% CI: 2.32%-4.23%) of all patients had a viral coinfection. We observed that peritoneal dialysis, patients submitted to a surrogated dialysis center and living with a close relative with prior hepatitis infection were associated conditions to had any viral coinfection. Our study suggests that infectious viral diseases still have a high prevalence in patients undergoing dialysis in any modality in Mexico.

Our results provide and epidemiological assessment of HBV, HCV, and HIV infection in Mexico. A multicenter study conducted by Paniagua R et al in 2010 reported that the prevalence of HBV and HCV in patients undergoing hemodialysis was 7.1 and 8.4%, respectively [9]. Another study by Calderón G et al found that prevalence within patients with multiple transfusions submitted to dialysis was 13.7% for HCV, 7% for HBV, and 1.7% for HIV. In Mexico, it has been showed that HBV outbreaks were frequent problems in hemodialysis patients since late 1990s [20]. Our estimations are lower compared with previous reports. However, the prevalence of HBV and HCV continues to be higher if we compare it with the national prevalence in general population. Rojo-Medina J. et al showed that the prevalence in general population of HBV in the years 2000 to 2012 within blood donors was 0.47% to 0.15%, while Carnalla M. et al showed that for HCV the national prevalence was 0.23% in the year 2018 [21, 22]. Furthermore, a pooled epidemiological report performed by the Pan-American Health Organization mentions that within Latin-American population, HBV yields a prevalence of 0.33% and HCV of 0.73%, respectively [23]. All the above-mentioned reports also demonstrated that the prevalence of HBV and HCV is higher within men, which is consistent with our estimations. Regarding HIV, diverse organization have sought to assess the national prevalence in our country. We found in our sample that HIV prevalence is similar for the estimations reported in general population [24, 25]. Overall, our results are consistent with previous reports that demonstrate that although the prevalence of HBV and HCV had decreased within patients submitted to dialysis, it still of high burden compared with the general population in Mexico [26].

Regarding the associated factors for developing the viral coinfections, it has been reported that there are various practices related to the individual and the treatment center that could influence the having coinfection diseases. It has been studied that a history of transfusion, use of illegal intravenous substances, risky sexual practices, lack of vaccination, and high-exposure groups (E.g., sex workers, prison inmates, unprotected health care personnel) are associated factors for acquiring HBV or HCV [27, 28]. Moreover, a recent multicenter study, demonstrate that within multiple centers in Europe, patients diagnosed with HCV coinfection were allocated within specialized centers that treated only patients coinfected with HCV [29]. Our results show that surrogated dialysis centers and being submitted to peritoneal dialysis units were associated factors for any viral coinfections. During our study period, the IMSS had a mandatory policy that all previously known hemodialyzed coinfected subjects were treated within this surrogated units, which could be the reason why we observed a higher rate of coinfection among these units. Furthermore, we also demonstrate that most of our prevalent cases were diagnosed within the last decade, which could be interpreted as patients could have better survival compared to patients treated before 2000’s. Regarding peritoneal dialysis, these patients are generally considered of low risk for viral infections, and the fact that we observed that this RRT modality represented an associated factor may be due to the lack of screening once the patient initiate the therapy which could be the reason why we observe a higher rate of coinfection within this modality. Overall, we found that living with a coinfected case was an individual associated factor for having any coinfection, which should raise awareness for screening the relatives of the patients that are submitted for any RRT to diminish the onset coinfections diseases. The lack of association with traditional factors related to viral coinfections may be due to several reasons: the increase in clinical awareness of epidemiological surveillance, mandatory screening strategies for viral coinfection within each dialysis unit, increase in hygiene and sanitization policies, removal of recurrent blood transfusions, among other factors that have been modified during time to provide a better clinical management for patients submitted to RRT.

Although several efforts have been made to assess the prevalence of the disease, there are still a lack of information within diverse vulnerable groups, including the incidence estimation of new infectious cases within patients submitted to hemodialysis, which is an area of opportunity for further research. With our results, we emphasize the urgent need to raise awareness of these diseases since the recent introduction of antiviral therapy against HCV in our institution, along with strengthening screening strategies to mitigate the impact of HBV, HCV, and HIV on population living with CKD in Mexico and Latin America.

### Strengths and limitations

Our study provides valuable epidemiological evidence regarding the prevalence and associated factors for HCV, HBV and HIV in patients submitted to dialysis across 21 units from the Mexican Institute of Social Security in Mexico. Additionally, this is one of the most recent refresh in the literature regarding the prevalence of viral coinfection of HBV, HCV, and HIV within RRT population living in Mexico. Nevertheless, we have some limitations to acknowledge. First, our study was designed as a cross-sectional recruitment of patients who attended in dialysis units at the State of Mexico, which may be bias toward the central urban region of Mexico and may not be representative towards all the regions in our country. Second, although our sample size is considerably higher compared with other studies performed in our country, the proportion of coinfected cases is low and could not allow us to perform appropriate stratifications by sociodemographic and clinical characteristics. Third, we were unable to assess serological testing within 1,090 subjects, because we were unable to recover the printed serological testing which overall represent a potential bias. Fourth, we were unable to estimate an incidence rate of viral coinfection, leading to future areas of research. Finally, our questionnaires were applied to patients who voluntarily participated in our recruitment as well as the questionnaire of clinical facility was applied to the chief responsible to each dialysis, which could be interpreted as a selection bias of not reporting undesirable practices.

## Conclusion

We observed that the prevalence of coinfection of HCV and HBV has decreased within recent years within patients submitted to hemodialysis but remains high compared with general population. Regarding HIV prevalence, we observed similar estimations compared to national assessments. Associated factors for any viral coinfection were peritoneal dialysis, being submitted to a surrogated dialysis center and living with a close relative with prior hepatitis infection. Overall, our study provides crucial information that demonstrate that HCV, HBV, and HIV continues of a problem of concern within patients submitted to dialysis, and therefore, should raise awareness within health authorities to strength screening strategies to the patients in any RRT modality and their relatives to promote better care practices within population living with CKD in Mexico.

## Supporting information

S1 File (DOCX)

## Abbreviations

CI: Confidence Interval
HBV: Hepatitis B Virus
HCV: Hepatitis C Virus
HIV: Human Immunodeficiency Virus
IMSS: Mexican Institute of Social Security
RRT: Renal Replacement Therapy
