# Supplementary material for: Prevalent coinfection and associated factors for Hepatitis B, Hepatitis C, and Human Immunodeficiency Virus in patients submitted to renal replacement therapy: A cross-sectional study of 21 dialysis units in the State of Mexico
Source: PLoS One. 2022 Dec 1;17(12):e0275238. doi: 10.1371/journal.pone.0275238 (PMC9714696; doi:10.1371/journal.pone.0275238)
Supplement: S1 File — (DOCX) [file pone.0275238.s001.docx]

**SUPPLEMENTARY MATERIAL**

*Sample Size Estimation*

We estimated a sample size according to the methods reported by Charan J et. al. [1]. For convenience, we used the “epi.sssimpleestb” function from the epiR package. We estimated a sample size for a binary outcome (prevalence) using a simple random sampling. We assumed an almost perfect sensitivity and specificity (>99%) for each diagnostic test for HBV, HCV, HIV, and a 95% confidence interval.

For HBV, we estimated a prevalence to be in the order of 8.4% (95% CI 5.5-11.2) based on the previous work published by Paniagua R et. al [2].

For HCV, we estimated a prevalence to be in the order of 6.7% based on the previously work published by Méndez-Sánchez N [3].

The relative error was derived from the relative error ($\varepsilon$), which is the maximum difference between the estimate and the unknown population value expressed in relative terms, was set to be of 0.33. The relative error was derived from the formula:

$\varepsilon=\frac{Upper or Lower Limit-Prevalence \left( \% \right)}{Relative prevalence}=\frac{11.2-8.4}{0.84}=0.33$

We required to test a minimum of 429 patients to estimate a prevalence for HBV.

The output of the R code is appended:

>library(epiR)

>epi.sssimpleestb(Py = 0.084, epsilon = 0.333, error = "relative", se = 0.99, sp = 0.99, nfractional = FALSE, conf.level = 0.95)

>429

For HBV infection, we required 562 patients.

The output of the R code is appended:

>library(epiR)

> epi.sssimpleestb(Py = 0.067, epsilon = 0.333, error = "relative", se = 0.99, sp = 0.99, nfractional = FALSE, conf.level = 0.95)

[1] 562

**Missing Variables**

We assessed and imputed missing biochemical values using multiple chained equations under the assumption that data was missed completely at random using the *mice* R Package (Version 3.14.0) (13). We multiply 5 imputed datasets for a maximum of 5 iterations combined using Rubin's rules. We present the missing data percentage (A), the density histogram (B), and summary statistics (C) of original and imputed variables.

**Training, capacitation of nursing personnel and validation of questionnaire.**

We trained 9 nurses to assess the clinical questionnaire in our studied population. A 5 hrs. debrief session was applied before the initiation of the study, where we briefly explained the epidemiology, physiopathology, diagnosis criteria of HBV, HCV, and HIV. Then, we explained the objectives within our protocol, as well as the rationale of our clinical questionnaire. The chief of each medical dialysis unit supervised and solve any medical questions of each interviewed patients when appropriated. A pilot study of the 5 questionnaires developed was carried out to estimate the approximate time in the application of each instrument and to train the personnel who will apply them. This requires 10% of the total sample. Within our clinical questionnaire, we performed the CRONBACH’S ALPHA resulting in an overall analysis of 0.701 with 18 degrees of freedom.

**Clinical and treatment facility questionnaires**

The full questionnaires used in this study are available within the following GitHub Repository:

Clinical questionnaire: <https://github.com/neftalivilla/hepatitis_dialisis/blob/main/Clinical_Questionaire.pdf>

Familiar questionnaire: <https://github.com/neftalivilla/hepatitis_dialisis/blob/main/Family_Questionaire.pdf>

Treatment facility questionnaire: <https://github.com/neftalivilla/hepatitis_dialisis/blob/main/Treatment_Facility_Questionaire.pdf>

**Supplementary Table 1:** Medication and biochemical characteristics of patients submitted to renal replacement therapy from the Metropolitan Area of Mexico

| **Parameter** | **All-Population**  **(n=1,304)** | **Without Any-Coinfection**  **(n=1,265)** | **Any-Viral Coinfection**  **(n=39)** | **P value** |
| --- | --- | --- | --- | --- |
| Erythropoietin use (%) | 993 (76.15) | 962 (76.05) | 31 (79.49) | 0.705 |
| CCBs use (%) | 303 (23.24) | 291 (23) | 12 (30.77) | 0.338 |
| Beta-blockers use (%) | 127 (9.74) | 123 (9.72) | 4 (10.26) | 0.981 |
| Thiazides use (%) | 160 (12.27) | 159 (12.57) | 1 (2.56) | 0.083 |
| Loop Diuretics use (%) | 224 (17.18) | 217 (17.15) | 7 (17.95) | 0.981 |
| ACEI use (%) | 181 (13.88) | 178 (14.07) | 3 (7.69) | 0.342 |
| Alpha Blockers use (%) | 124 (9.51) | 119 (9.41) | 5 (12.82) | 0.583 |
| ARBs use (%) | 418 (32.06) | 408 (32.25) | 10 (25.64) | 0.386 |
| Iron Supplement Intake (%) | 390 (29.91) | 385 (30.43) | 5 (12.82) | 0.025* |
| Hypoglycemiant use (%) | 101 (7.75) | 98 (7.75) | 3 (7.69) | 0.981 |
| Multivitamins use (%) | 811 (62.19) | 785 (62.06) | 26 (66.67) | 0.621 |
| Hemoglobin (gr/dl) | 9.7 (8.3-11.3) | 9.4 (7.9-10.9) | 9.8 (8.35-11.05) | 0.288 |
| Calcium (mg/dl) | 8.7 (8-9.4) | 8.5 (7.8-9.1) | 8.6 (8-9.5) | 0.222 |
| Sodium (mg/dl) | 139 (136.6-141.2) | 140 (137.6-142) | 139.2 (137.2-141.6) | 0.477 |
| Potassium (mg/dl) | 4.8 (4.2-5.4) | 5 (4.5-5.6) | 5 (4.5-5.7) | 0.978 |
| Phosphorus (mg/dl) | 5.4 (4.1-6.77) | 5.3 (4-6.6) | 4.7 (4-5.8) | 0.090 |
| Creatinine (mg/dl) | 10.4 (7.9-13.5) | 9.45 (7.47-11.89) | 9.71 (7.8-12.2) | 0.696 |
| Urea (mg/dl) | 118.75 (93.62-149) | 120 (97-147.2) | 123 (110-154) | 0.205 |
| Albumin (mg/dl) | 3.7 (3.4-4) | 3.8 (3.5-4.1) | 3.9 (3.6-4.1) | 0.378 |
| Total Bilirubin (mg/dl)  **n=107** | 0.4 (0.3-0.59) | 0.6 (0.4-0.8) | 0.38 (0.37-0.39) | 0.063 |
| Direct Bilirubin (mg/dl) **n=107** | 0.2 (0.11-0.21) | 0.2 (0.16-0.3) | 0.09 (0.08-0.1) | <0.001* |
| Indirect Bilirubin (mg/dl) **n=106** | 0.23 (0.16-0.38) | 0.4 (0.2-0.8) | 0.29 (0.28-0.29) | 0.306 |
| Phosphatase Alkaline (mg/dl) **n=125** | 116 (85-165) | 133.5 (103.25-177.02) | 106 (87-338.5) | 0.551 |
| Lactic Dehydrogenase (mg/dl)  **n=109** | 368 (255-464) | 304 (172-426.5) | 195 (187-198) | 0.157 |

Footnotes: For total, direct and indirect bilirubin, phosphatase alkaline and lactic dehydrogenase we reported the available variables.

**Supplementary Table 2:** Characteristics of the centers of dialysis in the eastern zone of Mexico.

| **Parameter** | **All-Centers (n=21)** |
| --- | --- |
| Peritoneal Dialysis (%) | 10 (47.62) |
| Hemodialysis (%)  Reutilization of Filters (%) | 11 (52.38)  8 (72.7%) * |
| External RRT (%) | 13 (61.9) |
| Annually attended patients (IQR) | 524 (248-783) |
| Use of Disposable Material (%) | 19 (90.48) |
| Serology Before RRT (%) | 15 (71.43) |
| Monthly Fumigation (%) | 14 (66.67) |
| Periodic Cleaning of Facilities (%) | 19 (90.48) |
| Isolation Mandates (%) | 21 (100) |
| HBV Vaccination Policy (%) | 20 (95.24) |
| 1 Dose (%) | 2 (9.52) |
| 2 Dose (%) | 11 (52.38) |
| 3 Dose (%) | 4 (19.05) |
| 4 Dose (%) | 2 (9.52) |

*Footnote*: * = Estimation only for 11 hemodialysis centers.
